# PM_2.5_ Pollution: Health and Economic Effect Assessment Based on a Recursive Dynamic Computable General Equilibrium Model

**DOI:** 10.3390/ijerph16245102

**Published:** 2019-12-13

**Authors:** Keyao Chen, Guizhi Wang, Lingyan Wu, Jibo Chen, Shuai Yuan, Qi Liu, Xiaodong Liu

**Affiliations:** 1 National Climate Center, China Meteorological Administration, Beijing 100081, China; chenky@cma.gov.cn; 2 School of Mathematics and Statistics, Nanjing University of Information Science & Technology, Nanjing 210044, China; wulingyan14@126.com (L.W.); chenjibo@nuist.edu.cn (J.C.); yuanshuaicrystal@163.com (S.Y.); 3 Shandong Beiming Medical Technology Ltd., Jinan 250000, China; qrankl@163.com; 4 School of Computing, Edinburgh Napier University, Edinburgh EH10 5DT, UK; x.liu@napier.ac.uk

**Keywords:** haze pollution, genetic algorithm, exposure-response model, computable general equilibrium model, health effects

## Abstract

At present particulate matter (PM_2.5_) pollution represents a serious threat to the public health and the national economic system in China. This paper optimizes the whitening coefficient in a grey Markov model by a genetic algorithm, predicts the concentration of fine particulate matter (PM_2.5_), and then quantifies the health effects of PM_2.5_ pollution by utilizing the predicted concentration, computable general equilibrium (CGE), and a carefully designed exposure–response model. Further, the authors establish a social accounting matrix (SAM), calibrate the parameter values in the CGE model, and construct a recursive dynamic CGE model under closed economy conditions to assess the long-term economic losses incurred by PM_2.5_ pollution. Subsequently, an empirical analysis was conducted for the Beijing area: Despite the reduced concentration trend, PM_2.5_ pollution continued to cause serious damage to human health and the economic system from 2013 to 2020, as illustrated by various facts, including: (1) the estimated premature deaths and individuals suffering haze pollution-related diseases are 156,588 (95% confidence intervals (CI): 43,335–248,914)) and six million, respectively; and (2) the accumulated labor loss and the medical expenditure negatively impact the regional gross domestic product, with an estimated loss of 3062.63 (95% CI: 1,168.77–4671.13) million RMB. These findings can provide useful information for governmental agencies to formulate relevant environmental policies and for communities to promote prevention and rescue strategies.

## 1. Introduction

The acceleration of urbanization, extensive development of the economy, and ignorance of environmental protection can cause an astonishing deterioration of atmospheric quality. Many epidemiological studies have reported harmful health effects (e.g., respiratory diseases, cardiovascular disease, and other deadly diseases) due to air pollution [1,2,3,4], especially the pollution by small particles like fine particulate matter (PM_2.5_).

In China, due to the rapid development experienced over the past three decades, haze pollution has increased to an intolerable level, which accordingly concerns not only the government and local officials, but also all citizens of the country. Specifically, mobile vehicles (on the road: automobiles and motorcycles; non-road: aircrafts, marine engines, ocean vessels, and recreation vehicles) emit ozone, haze pollution, and air toxins, and are the main sources of ambient PM_2.5_ concentrations.

### 1.1. Existing Studies and Study Methods for Health Consequences

Kan et al. [5] used a generalized additive model to analyze the effects of PM_2.5_ on mortality in Shanghai, and their results demonstrated that every 10 μg/m^3^ increase in the 2-day moving average concentration of PM_2.5_ would cause an increase in total mortality rate by 0.36% (95% confidence intervals (CI): 0.11–0.61%)), and increases in cardiovascular and respiratory mortality rates, by 0.41% (95% CI: 0.01–0.82%) and 0.95% (95% CI: 0.16–1.73%), respectively. According to a *Lancet* study [6], the mortality due to the presence of toxic chemicals or compounds for China is an order higher than the combined figures of traffic injuries and Human Immunodeficiency Virus/Acquired Immuno Deficiency Syndrome (HIV/AIDS) infections, and has become a leading cause of death. Xie et al. [7] assessed the health hazards and economic damage for the Beijing area for a period of time with high-level PM_2.5_ exposure (January 10th to 15th, 2013), and employed the Poisson regression model and environmental valuation method. Their results showed that the health-related economic losses alone was estimated as high as 489 (95% CI: 204–749) million RMB.

A new study released in *Nature* [8] found that outside toxic chemicals or compounds, largely from PM_2.5_, led to 3.3 (95% CI: 1.6–4.8) million premature deaths worldwide yearly, prevailing in Asia. Astonishingly, 1.357 million of these happened in China alone. For the United States, the corresponding number is 55,000.

Kioumourtzoglou et al. [9] estimated the impacts of long-term exposure of PM_2.5_ on survival in 81 cities across the United States and found a positive association between long-term exposure to PM_2.5_ and all-cause mortality, with a hazard ratio (hazard ratio is the ratio of two hazard rates and can be abbreviated as HR – it reflects the difference between the two hazard rates) of 1.11 (95% CI: 1.01–1.23) per 10 µg/m^3^ increase in the annual PM_2.5_ concentration. Chen et al. [10] utilized the Cox proportional hazards model to determine the influence of exposure to PM_2.5_ on survivors of myocardial infarction in Ontario, Canada. Their results show that for each 10 μg/m^3^ increase in PM_2.5_, the adjusted HR of cardiovascular disease mortality, ischemic heart disease mortality, and acute myocardial infarction mortality were 1.35 (95% CI: 1.09–1.67), 1.43 (95% CI: 1.12–1.83), and 1.64 (95% CI: 1.13–2.40) respectively, which was even higher than in Kioumourtzoglou’s study for these deadly heart diseases.

### 1.2. Existing Methods on Health-Related Economic Effects

At present, the dominant methods to estimate economic costs due to air pollution include the human capital approach (HCA is a method to use the loss of income to value the cost of premature death due to pollution), cost of illness (COI is a method to evaluate the economic loss caused by environmental pollution to human health and labor capacity), and contingent valuation method (CVM is a survey-based method for assessing the value of non-market goods and services). Zhang et al. [11] applied HCA to calculate economic losses due to air pollution in Lanzhou for 2006, and their result showed a loss of 474 million RMB. Fan [12] applied the amended HCA to analyze the economic loss due to health damages caused by SO_2_, NO_2_, and PM_10_ in Beijing City, China in 2012, and his result showed a total loss of 768.58 million RMB for 2012. Zhao et al. [13] evaluated the economic impact from illness and, by utilizing amended HCA, premature death incurred by PM_10_ pollution in Beijing for 2012, and they found that economic loss was 583.02 million RMB. Othman et al. [14] assessed the economic value of haze pollution in the state of Selangor, Malaysia by COI, and the results showed an annual loss of $91,000 due to the inpatient health impact of haze. Lee et al. [15] used CVM to investigate the reduction in value of a statistical life (VSL) due to the air pollution risk in the Seoul metropolitan city of South Korea, $485,000 (95% CI: 398,000–588,000) in VSL, and to estimate the damage cost due to risk of PM_2.5_ inhalation, about $1057 million per year for acute exposure and $8972 million per year for chronic exposure. Recently, Akhtar et al. [16] used CVM to estimate individual’s willingness to pay (WTP) for air quality control in an urban area of Lahore (India), and the results gave a mean WTP per person per year ($118.32) for a 50% reduction in air pollution.

Huang et al. [17] monetized the morbidity and mortality effects using COI, amended HCA, and CVM. The results showed that in 2006 the total economic loss of PM_10_ pollution in the Pearl River Delta region (China) was 29.21 billion RMB using CVM and COI, and was 15.51 billion RMB using amended HCA and COI.

Yang et al. [18] calculated used HCA and COI to calculate the economic cost associated with the human health damage from air pollution before and after the clean energy reform, a program launched in Lanzhou (China) in 2005. The program aimed to reform of the buses, taxis, and coal-fired boilers, and Yang et al. found that economic losses were 0.86 and 1.35 billion RMB in 2003 and 2008 respectively.

### 1.3. Gap in the Existing Studies and Our Approach to Narrow the Gap

The results reviewed in previous Section 1.1 and Section 1.2 only measured the economic burden of disease rather than gross domestic product (GDP), and the study methods cannot simulate the intricate chain effect between industry sectors. Hence, in previous work, an input-output (I-O) model [19] and a static computable general equilibrium (CGE) model [20] have been applied to the field of economic loss assessment for haze pollution to resolve these two shortcomings.

However, the I-O model also has many limitations (e.g., linearity and absence of prices) [21], whereas the static CGE does not give a consideration to the persistence of disaster effects [22]. Therefore, this paper intend to resolve this by employing the dynamic CGE. In the past two years, dynamic CGE approaches have been applied to the field of haze pollution. Ma et al. [23] constructed a dynamic CGE model to evaluate SO_2_ emission on the economy under the haze pollution. Results showed that the SO_2_ emissions would drop 30–40% by 2030. Xu et al. [24] proposed a dynamic CGE model to assess the impact of different coal resource tax rates on haze pollution. Results showed that increasing the coal resources tax can reduce haze pollution, so increment of tax rates could be an effective choice for decreasing PM_2.5_. In current haze pollution research, the use of dynamic CGE models is mostly based on carbon emissions and SO_2_ emissions. The innovation of this paper is to select PM_2.5_ as a representative indicator of haze and establish a dynamic CGE model to assess the economic impacts and health impacts of haze. PM_2.5_ is the main culprit in causing haze; hence, selecting PM_2.5_ will make the evaluation results more realistic.

In the remainder of the paper, Section 2 develops the genetic algorithm (GA)-grey Markov model (GM), exposure-response (E-R) functions and recursive dynamic CGE model. The CGE model can take into account the influence of PM_2.5_ pollution on different economic entities and different industrial sectors. The dynamic CGE model can measure the long-term economic damage incurred by PM_2.5_. Section 3 applies the models established in Section 2 to calculate the economic and health losses by haze pollution, including the long-term trend of economic effects. In Section 4, we discuss and elaborate some limitations of the proposed method. In Section 5, the conclusions are drawn.

## 2. Materials and Methods

In this section, the basic principles, the mathematical expressions and characteristics of the GA-grey Markov model, E-R functions, and recursive dynamic CGE model are elaborated.

### 2.1. GA-Grey Markov Model

#### 2.1.1. Model Building

The modeling steps of the GA-grey Markov model are described as follows:

*Step 1*: Establishing a GM (1,1) model [25]:

(1) $$X^{\left(0\right)}=\left\{X^{\left(0\right)}\left(1\right),\;\ldots ,X^{\left(0\right)}(k),\ldots ,X^{\left(0\right)}\left(n\right)\right\}\;,n\geq 4$$

Let this be the original sequence (e.g., pollution concentration data, economic data, or other series). A new sequence is generated by the accumulated generating operator (AGO):

(2) $$X^{\left(1\right)}=\left\{X^{\left(1\right)}\left(1\right),\ldots ,X^{\left(1\right)}\left(k\right),\ldots ,\;X^{\left(1\right)}\left(n\right)\right\}\;,\;n\geq 4$$

where $X^{\left(1\right)}\left(k\right)=\sum_{i=1}^{k}X^{\left(0\right)}\left(i\right)\;\left(k=1,\;2,\;\ldots ,\;n\right)$. The grey generated model based on Equation (2) is given by a first-order differential equation:

(3) $$\frac{dX^{\left(1\right)}(t)}{dt}+aX^{\left(1\right)}(t)=\mu \;$$

where a and μ denote the development coefficient and the grey control parameter, respectively. Equation (3) is referred as the first order grey differential equation and denoted by GM (1,1).

The solution of Equation (3) with system parameters is determined by the initial condition $X^{\left(1\right)}(1)=X^{\left(0\right)}(1)$ and least-squares method. Equation (4) is a step in the theoretical derivation of a GM (1,1) model, which is obtained by introducing $\tilde{\alpha }$ into Equation (3):

(4) $${\tilde{X}}^{\left(1\right)}\left(t\right)=\left(X^{\left(1\right)}\left(1\right)-\mu /a\right)\cdot e^{-a\left(t-1\right)}+\mu /a\;,\;\tilde{\alpha }=\left(\begin{array}{c} a\\ \mu \end{array}\right)\;={\left(B^{T}B\right)}^{-1}B^{T}Y_{n}$$

where:

(5) $$\;Y_{n}=\left(\begin{array}{c} X^{\left(0\right)}\left(2\right)\\ X^{\left(0\right)}\left(3\right)\\ \vdots \\ X^{\left(0\right)}\left(n\right) \end{array}\right),\;B=\left(\begin{array}{cc} -\frac{1}{2}\left[X^{\left(1\right)}\left(1\right)+X^{\left(1\right)}\left(2\right)\right] & 1\\ -\frac{1}{2}\left[X^{\left(1\right)}\left(2\right)+X^{\left(1\right)}\left(3\right)\right] & 1\\ \vdots & \vdots \\ -\frac{1}{2}\left[X^{\left(1\right)}\left(n-1\right)+X^{\left(1\right)}\left(n\right)\right] & 1 \end{array}\right)$$

With the inverse AGO, the predicted values of GM (1,1) are obtained by Equation (6):

(6) $${\tilde{X}}^{\left(0\right)}\left(k+1\right)=\left\{ \begin{array}{ll} X^{\left(0\right)}\left(1\right) & k=0\\ {\tilde{X}}^{\left(1\right)}\left(k+1\right)-{\tilde{X}}^{\left(1\right)}\left(k\right)=\left(1-e^{a}\right)\cdot \left(X^{\left(0\right)}\left(1\right)-\mu /a\right)\cdot e^{-ak} & k=1,\;2,\;\ldots ,\;n-1 \end{array}\right.\;$$

Furthermore, the residual series are written as:

(7) $$\tilde{e}\left(k\right)=X^{\left(0\right)}\left(k\right)-{\tilde{X}}^{\left(0\right)}\left(k\right),\;k=1,\;2,\;\ldots ,\;n\;$$

*Step 2*: Dividing the states of $\tilde{e}$ based on the grey relational grade test can reflect the similarity degree theory [26]. Ensure that each state has at least one real value and every real value is assigned to a certain state. The i−th state is signified as:

(8) $$d_{i}=\left[l_{i},\;u_{i}\right),\;i=1,\;2,\;\ldots ,\;r\;$$

Suppose $\tilde{e}\left(t\right)\in d_{i}$, the predicted value is:

(9) $${\hat{X}}^{\left(0\right)}\left(t+k\right)={\tilde{X}}^{\left(0\right)}\left(t+k\right)+\sum_{j=1}^{r}p_{ij}^{\left(k\right)}h_{j},i,\;j=1,\;2,\ldots ,\;r;\;k=1,\;2,\;\ldots ,\;n-1\;$$

where $p_{ij}^{\left(k\right)}$ too big is the k-step transition probability [27]; and $h_{j}$ too small is defined as:

(10) $$h_{j}=\lambda_{j}l_{j}+\left(1-\lambda_{j}\right)\;u_{j},\;j=1,\;2,\;\ldots ,\;r\;$$

where $\lambda_{j}\in \left[0,1\right]$ is usually set to 0.5 in the traditional grey Markov model, which may not be optimal. Scale all equations to the same size as the text.

*Step 3*: Solving the optimal value of λ by GA [28]. The mean squared error (MSE) below is used as the fitness function:

(11) $$MSE=\sum_{k=1}^{n}e^{2}\left(k\right)/n\;$$

where $e\left(k\right)=X^{\left(0\right)}\left(k\right)-{\hat{X}}^{\left(0\right)}\left(k\right)$. Align with text.

#### 2.1.2. Model Checking

The residual test measures the error between the prediction sequence and the original sequence by using the average relative error. First, define the absolute error as:

(12) $$\Delta^{\left(0\right)}\left(i\right)=\left|X^{\left(0\right)}\left(i\right)-{\hat{X}}^{\left(0\right)}\left(i\right)\right|,\;i=1,\;2,\;\ldots ,\;n\;$$

The average relative error is written as:

(13) $$\bar{\Phi }=\frac{1}{n}\sum_{i=1}^{n}\frac{\Delta^{\left(0\right)}\left(i\right)}{X^{\left(0\right)}\left(i\right)}\;,\;i=1,\;2,\;\ldots ,\;n\;$$

The grey relational grade test reflects the similarity degree between the predicted sequence (see Equation (9)) and the original sequence (see Equation (1)), and is defined as the mean value of the relational coefficients, that is:

(14) $$\gamma =\frac{1}{n}\sum_{i=1}^{n}\frac{\underset{1\leq i\leq n}{\min}\Delta^{\left(0\right)}\left(i\right)+\rho \underset{1\leq i\leq n}{\max}\Delta^{\left(0\right)}\left(i\right)}{\Delta^{\left(0\right)}\left(i\right)+\rho \underset{1\leq i\leq n}{\max}\Delta^{\left(0\right)}\left(i\right)},\;i=1,\;2,\;\ldots ,\;n\;$$

where ρ∈(0, 1) is a distinguishing coefficient, where the smaller value of ρ is, the larger distinguished ability is, so the default value of ρ is 0.5. γ is the value of the grey relational grade, ranging from 0 to 1. It equals 1 if the predicted sequence and the original one are identically matched.

The posteriori error test describes the statistical properties of a residual sequence according to the posteriori error ratio and small error probability. These two indicators are respectively defined as follows:

(15) $$C=S_{2}/S_{1}\;$$

(16) $$P=p\left\{\;\left|\Delta^{\left(0\right)}\left(i\right)-{\bar{\Delta }}^{\left(0\right)}\right|<0.6745S_{1}\right\}\;,i=1,\;2,\;\ldots ,\;n\;$$

where $S_{1}$ and $S_{2}$ are the standard deviations of original sequence and absolute error sequence, respectively; ${\bar{\Delta }}^{\left(0\right)}$ is the mean value of absolute errors.

### 2.2. E-R Functions

The E-R functions are often applied to the quantitative analysis of health risks. According to the studies by Pascal et al. and Maji et al. [29,30], E-R functions can be formulized by:

(17) $$E=P\cdot I\cdot \left\{1-\frac{1}{e^{\beta \left(C-C_{0}\right)}}\right\}$$

where the formula calculates the reduction (E) in the number of illness or deaths along with the reduction of PM_2.5_ concentration; P denotes the size of the exposed population; I is the actual incidence of the population; β denotes the E-R coefficient; C represents the actual PM_2.5_ concentration; and $C_{0}$ is the baseline concentration. Schwartz et al. [31] found no evidence of a threshold in the association between PM_2.5_ and daily deaths. Therefore, we set $C_{0}$ to 0 μg/m^3^.

### 2.3. Recursive Dynamic CGE Model

The CGE model is firstly recalled and then the paper focused on design of a dynamic CGE.

#### 2.3.1. CGE Model

The computable general equilibrium (CGE) model employs real data reflecting economic activities to explore the reaction of economic systems changes in policy and technology. The model contains necessary mathematical representations of an economy covering economic and behavioral operations of manufactures, suppliers, consumers, federal and local governments, investors, and exporters. All participants have their respective underlying behaviors that determine their decisions [32].

Particularly, an advantage of the dynamic CGE model is to admit a cross-period study and extend the field of study, and this has become an indispensable tool for policy analyses. In addition, the CGE method is used to the long-time adoption in analyzing national economic policies, which also increases popularity in analyzing local policies. CGE model has also become the most influential method for analyzing local economic supporting policies. Hence, it is appropriate to apply the dynamic CGE model for assessing the long-term economic effects of the labor loss and medical expenditures in this paper.

#### 2.3.2. Dynamic CGE Model

The proposed CGE model depicts the combined impacts of the government policy and the market price. PM_2.5_ is incapable of circulating nation-wide and participating in the import and export trade. Hence, the CGE model is assumed in a closed economic condition without including other regions and enterprise accounts. Furthermore, the 42 sectors in Beijing’s input-output table in 2012 are merged into six sectors, including ‘Agriculture’, ‘Industry’, ‘Construction’, ‘Transportation, ‘Postal and telecommunication’, ‘Health services’, and ‘Other services’. The sets of activities and commodities are devoted as M and N respectively; m∈M, n∈N, m,n=1, 2, …, 6. The major relevant formulas are provided as follows (for a complete model, see Zhang [33]).

(1) Price module

(18) $$PA_{m}\cdot QA_{m}=PVA_{m}\cdot QVA_{m}+PINTA_{m}\cdot QINTA_{m}\;$$

where QA, QVA and QINTA stand for the quantity level, the quantity of value-added, and the quantity of aggregate intermediate input, respectively; and PA, PVA and PINTA are the corresponding prices respectively. Equation (18) implies that the income of each activity is fully exhausted by payments for value-added and intermediate inputs.

(2) Production module

(19) $$QA_{m}=\alpha_{m}^{q}{\left[\delta_{m}^{q}QVA_{m}^{\rho_{m}}+(1-\delta_{m}^{q})QINTA_{m}^{\rho_{m}}\right]}^{1/\rho_{m}},$$

(20) $$QVA_{m}=\alpha_{m}^{va}\cdot QLD_{m}^{\eta_{m}}\cdot QKD_{m}^{1-\eta_{m}},$$

where QLD and QKD denote the quantities of labor and capital, respectively; $\alpha^{q},\;\delta^{q}\;\mathrm{and}\;\rho$ are the efficiency parameter, share parameter, and substitution parameter of the constant elasticity of substitution (CES) function, respectively; and $\alpha^{va}\;\mathrm{and}\;\eta$ denote the efficiency parameter and elasticity coefficient of the Cobb–Douglas (C-D) production function, respectively. In Equation (19), the activity level represents a value-added CES function. The aggregate intermediate input by Equation (20) describes the added value with a C-D production function.

(3) Institution module

(21) YH=WL⋅QLS+WK⋅QKS 

(22) $$YG=\sum_{m}\left[tv_{m}(WL\cdot QLD_{m}+WK\cdot QKD_{m})\right]+ti\cdot YH$$

where YH and YG represent the incomes of residents and government, respectively; QLS and QKS, are the quantities of labor and capital supplied, respectively; WL and WK are the average prices of labor and capital, respectively; ti denotes the personal income tax rate; and $tv_{m}$ denotes the value-added tax rate. Equations (21) and (22) describe the income sources for residents and government, respectively.

(4) System module

(23) $$QQ_{n}=\sum_{m}QINT_{mn}+QH_{n}+QINV_{n}+QG_{n}$$

where QQ stands for the quantity of goods supplied to market; QINV is quantity of fixed investment; QINT is quantity of commodity as intermediate input to activity; and QH and QG denote the quantities of consumptions for residents and government, respectively. Equation (23) imposes an equality between quantities supplied and demand for commodity.

(5) Recursive dynamic module

(24) $$QLS_{m}^{t+1}=QLS_{m}^{t}\cdot \left(1+g_{L}\right)$$

(25) $$QKS_{m}^{t+1}=QKS_{{}^{m}}^{t}\cdot \left(1+g_{K}\right)$$

(26) $$\alpha_{t+1}^{q}=\alpha_{t}^{q}\cdot \left(1+g_{tfp}\right)$$

where $g_{L},\;g_{K}\;\mathrm{and}\;g_{tfp}$ represent the growth rates of labor, capital, and total factor production (TFP refers to the comprehensive productivity of each element of the production unit), respectively.

The dynamic component of the CGE model is used to link the equilibrium state in the current period to the equilibrium state in the next period, primarily including productive factors’ accumulation and technological progress. Equations (24)–(26) exhibit how labor, capital, and TFP are updated in every period.

## 3. Results

### 3.1. Data

#### 3.1.1. PM_2.5_ Concentration

Haze pollution will not only cause severe harm to people’s health, but also affect the operation of the entire economic system. As a result, haze pollution causes labor force losses and medical expenses to the residents. The health effects of haze pollution are assessed by Equation (17). This requires the use of pollutant concentrations to calculate labor force loss and medical expenses, which uses the proposed dynamic CGE model to assess economic impacts of haze pollution. Therefore, it is most representative to choose the PM_2.5_ concentration to assess the health and economic impacts of haze from the above literature review and model requirements.

Since 2013, the Beijing Municipal Environmental Protection Bureau has listed PM_2.5_ in the environmental monitoring index and released official annual concentration data. The annual PM_2.5_ concentration is obtained from the ‘Beijing Environmental Statement’ of each year, and is summarized in Table 1.

From 2013 to 2017, haze pollution surveillance was implemented in 74 cities during the first phase. The annual average concentration of PM_2.5_ was 57.6 μg/m^3^, which is significantly higher than the annual average concentration 8.37 μg/m^3^ for the United States (as measured at 455 sites in the country). From Table 1, for Beijing, the average PM_2.5_ concentration was at 77.4 μg/m^3^ during the period from 2013 to 2017, which is significantly higher the average 57.6 μg/m^3^ for the 74 cities.

#### 3.1.2. Population

The population data includes the exposed population, labor force and mortality. Here the exposed population stands for the actual population including any floating population in the region. Therefore, the resident population are identified as the exposed population. Further, it is assumed that the population is exposed to the average pollution level.

Beijing is the capital of China, the world’s second most populous city proper (6336 sq miles—urban: 528 sq miles and rural: 5808 sq miles) and the most populous capital city (21.2 million in 2013; see Table 2 below). The city, located in northern China, is governed as a direct-controlled municipality under the national government with sixteen urban, suburban, and rural districts.

In Table 2, the mortality is stable in Beijing. Therefore, this paper assumes that mortality rates (‰, deaths per one thousand persons) during 2017 to 2020 are maintained at the level of 2016. For the resident population and labor force, assuming that the annual growth rates for 2017–2020 are maintained at the average levels during 2013–2016, that is 1.23% and 2.46% per year, respectively. The relevant population data used in this paper is given in Table 2.

#### 3.1.3. Related Information of Health

Taking the data availability into consideration, the paper assumes the morbidity, the proportion of medical visits, and the per capita medical cost which are kept at the levels of 2013.

We chose health outcomes that can be quantitatively estimated, including all-cause mortality, hospital admissions (respiratory and cardiovascular), outpatient visits (pediatrics and internal medicine), acute bronchitis, chronic bronchitis, and asthma. Only the acute bronchitis, chronic bronchitis, and asthma patients without seeking medical services are considered to avoid repetitive computation. We suppose the proportion of medical visits is the same as the national level, which is 72.7% and this is derived from ‘An Analysis Report of National Health Services in China’. Table 3 shows the E-R coefficients and their 95% CIs, the incidence and the delayed work days.

Furthermore, the excess medical expenditure of PM_2.5_ pollution is calculated. Due to data availability, only the medical cost of outpatients and inpatients are considered, and per capita cost data are used. According to the Beijing health service development statistical bulletin, in 2013, the per capita medical expenses of outpatients and inpatients are 393.3 RMB and 18,495.9 RMB, respectively.

#### 3.1.4. Social Accounting Matrix

As the fundamental part of developing a CGE model, a social accounting matrix (SAM) captures all the income and expenditure flows of the whole social-economy activities in the form of a two-dimensional table. In this paper, a SAM is constructed based on the 42 departments’ input–output tables in Beijing in 2012, which can be seen in Table 4 with recently available data. In addition, world account, other regions account, and enterprise account are not included in the SAM. The reason is that the CGE model in this paper is constructed in a closed economic condition and assumes the excess profit of any enterprise is equal to zero.

#### 3.1.5. Estimation Parameters of the CGE Model

*Population growth rate.* Zhang et al. [36] pointed out that, in Beijing, there will be no shortage of the labor force supply in the next ten years. Additionally, the impact on the labor input of the universal two-child policy, which was put into effect from 1 January 2016, and is expected to have some influence on the labor force after 2030 is not considered [37]. Based on the research results concerned, the growth rate of labor is taken as a value of the annual growth rate $g_{L}=2.46\%$, during 2013 to 2016.

*Capital growth rate.* Xu et al. [38] estimated Chinese capital stock by sector and region for 1978–2002 using the perpetual inventory approach. Their results indicated that the capital stock annual growth rate of Beijing was about 14.2%. Sun et al. [39] found that the annual growth rate of capital stock of Beijing was 13.4% during 1978–2008. Based on these two estimates, growth rate of capital stock is set to 13%.

*TFP growth rate.* Using DEA-Malmquist method, Wang and Fan [40] measured the TFP of 30 provinces in China for 1998–2012, and the average growth rate in Beijing was estimated to be 2%. Qi and Ma [41] estimated the average TFP growth rate of Beijing from 1978 to 2008 to be 2.07% with the Solow residual method. In view of the current development situation of Beijing, the growth rate of TFP is set to 2%.

*Estimation of parameters of production function.* The parameters of the production functions are estimated by econometric methods [42], with their high reliability based on the statistical data for many years. The parameters of the production functions are subject to the assumptions of the dynamic CGE model. The assumption of the dynamic CGE model are written as follows: (1) commodities in all industries are used for consumption or intermediate use; (2) if residents are regarded as a whole group, the consumption functions can be same; (3) during the period of study, the depreciation rate of fixed investment in each sector remains unchanged.

For the CES production function, let:

(27) $$f\left(\rho \right)=\ln[\delta^{q}\cdot QVA^{\rho }+(1-\delta^{q})\cdot QINTA^{\rho }]$$

Then Equation (19) can be written as:

(28) $$\ln\left(QA\right)=\ln\left(\alpha^{q}\right)+f\left(\rho \right)/\rho$$

Using the Maclaurin formula, f(ρ) can be extended. After disregarding the terms of third and higher orders, we have:

(29) $$\ln\left(QA\right)=u_{0}+u_{1}\cdot \ln\left(QVA\right)+u_{2}\cdot \ln\left(QINTA\right)+u_{3}\cdot {\left[\ln(QVA/QINTA)\right]}^{2}+\varepsilon$$

where $u_{0}=\ln\left(\alpha^{q}\right)$, $u_{1}=\delta^{q}$, $u_{2}=1-\delta^{q}$, $u_{3}=0.5\rho \cdot \delta^{q}\cdot (1-\delta^{q})$, and ε is the error term. It is assumed that ε obeys a normal distribution.

For the C-D production function, take the natural logarithm of both sides of Equation (20):

(30) $$\ln\left(QVA\right)=v_{0}+v_{1}\ln\left(QLD\right)+v_{2}\ln\left(QKD\right)+\varepsilon$$

where, $v_{0}=\ln\left(\alpha^{va}\right)$, $v_{1}=\eta$, $v_{2}=1-\eta$, and ε is the error term, which obeys a normal distribution. QLD, QKD are independent of ε.

The parameters of Equations (29) and (30) can be estimated by least squares estimation according to the input–output table of Beijing during 1985–2012. The remaining parameters can be calibrated based on SAM.

### 3.2. PM_2.5_ Concentration Prediction

Based on the concentration data during 2013–2017, PM_2.5_ concentration of Beijing during 2018–2020 is predicted with the GA-grey Markov model.

We establish a GM (1,1) prediction model based on the original sequence, which is the annual concentration data of PM_2.5_ for 2013–2017 in Beijing. Then parameters are estimated and the GM (1,1) model is constructed as follows:

(31) $${\hat{X}}^{\left(1\right)}\left(k+1\right)=-783.6215e^{-0.1193k}+873.1215,\;k=0,\;1,\;\ldots ,\;4$$

Furthermore, the predicted value can be calculated by the inverse AGO using Equation (6). The range of the residual series is divided into three states: overestimation state, accuracy state, and underestimation state, denoted as $d_{1}=[-4,\;-2)$, $d_{2}=[-2,\;2)$, and $d_{3}=[2,\;4]$, respectively. The predicted results and the state division of the residual series can be seen from Table 5.

According to the state division results of Table 5, the 1-step transition probability matrix can be written as:

(32) $$P=\left(\begin{array}{ccc} 0 & 0 & 1\\ 1 & 0 & 0\\ 1/2 & 0 & 1/2 \end{array}\right)$$

Year 2013 has been selected as the base year. The predicted concentration of the following years has been conducted by the traditional grey Markov model and GA-grey Markov model respectively. The GA parameters are set as follows: population size = 30, termination generation = 300, crossover rate = 0.8, and mutation rate = 0.1. By optimizing the fitness function after 65 iterations, the optimal value of whitening coefficients is achieved, as (0.004, 0.568, 0.993). Table 6 shows the predicted results.

Then, we evaluated the predicted performance of these three models based on the evaluation indexes in Section 2.1.2 and MSE, and results are presented in Table 7.

As shown in Table 7, the prediction precision of the GA-grey Markov model is greatly improved compared with the first two methods, and the MSE also reduces to 6.873. With the GA-grey Markov model, the predicted results of PM_2.5_ concentration for Beijing in 2018–2020 are listed in Table 8.

The three prediction models all have a Small Error Probability of 1. From the perspective of the Posteriori Error Ratio, GM (1,1) is the best, the GA-grey Markov model is the second, and the grey Markov model is the worst. From each of the other three indicators, the GA-grey Markov model is the best.

### 3.3. Health Effects of PM_2.5_ Pollution

Health damages influence the national economic system in two aspects: first, pollution-related illness can result in a loss or decline of working capacity; and second, these illnesses can increase medical expenses. To expand the previous studies [43], the economic effects regarding labor force and medical expenses are estimated.

**Figure 1:**
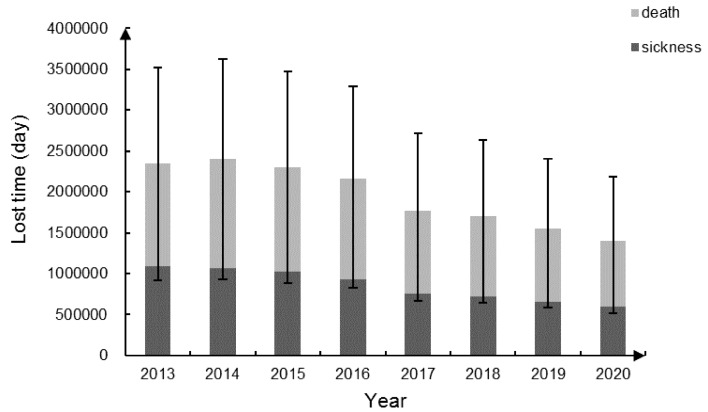
The lost time of the labor force caused by PM_2.5_ pollution in Beijing during 2013–2020.

Substituting E-R coefficients, exposed population, PM_2.5_ concentration and the incidence into Equation (17), the impact of PM_2.5_ pollution on population health can be calculated. According to the results, PM_2.5_ pollution could be seriously harmful to human health during 2013–2020 in Beijing, including 156,588 (95% CI: 43,335–248,914) premature deaths and 6,397,553 (95% CI: 3,056,828–9,381,570) cases of related diseases. Based on the Beijing Public Health and Population Health Status Report (edited by the Beijing Municipal People’s Government, People’s Medical Publishing House, 2014), labor force population (15–64 years old) accounts for 22.44% of all deaths. If assuming that this proportion remains unchanged during the study period, the loss of working days for the labor population who die or suffer from illness can be evaluated based on Table 3. Figure 1 shows the loss of working days and the corresponding 95% CI.

Furthermore, assuming 250 working days per person per year, the loss of working days is converted into the loss of labor quantity. The medical expenditure can be estimated based on the per capita medical expenses. Table 9 presents two conducting variables of the CGE model.

### 3.4. Economic Effects of PM_2.5_ Pollution

**Figure 2:**
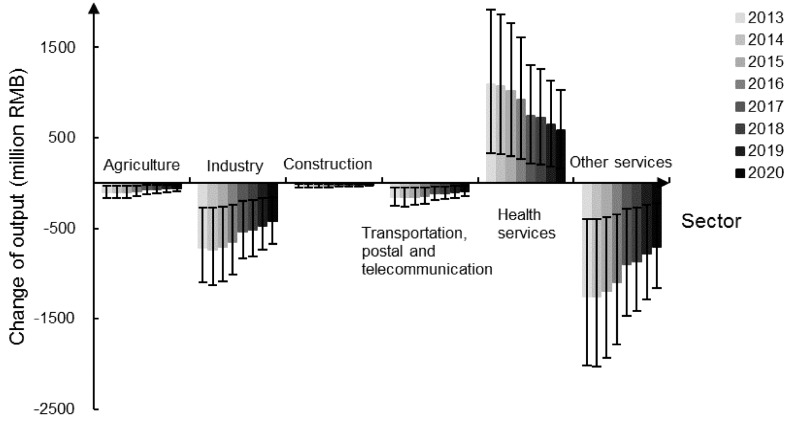
Sectors output changing in Beijing from 2013 to 2020.

Beijing’s PM_2.5_ pollution for 2013–2020 has been simulated, by introducing the labor loss and the medical expenditure into the dynamic CGE model. The main results are shown in figure and Figure 2.

Table 10 reflects the changes of macro-economic indicators relative to the baseline forecast, and these simulation results indicate that PM_2.5_ has a negative economic impact from macroscopic perspective. For one thing, the overall residents’ income level declines with the labor force shrinks, which implies less tax revenue. This may lower governmental revenues, with a corresponding reduction in government consumption under the request of neoclassical model. On the other hand, the increase of the medical expenditure implies a corresponding reduction of other products’ consumption. Due to a relatively smaller proportion that the medical and other health services take in the entire national economy (2.5% in 2016, Beijing Statistical Yearbook (2017)), their ability to drive the overall level of consumption is limited, and hence the overall level of residents’ consumption has declined.

The loss of labor input lowers the output level, and the increase of the medical expenditure leads to the drop in the overall consumption level, which means consumers’ reduced demand. The falling output and demand cause a drop in the regional GDP. Moreover, with a gradual control of haze pollution, the losses present a decreasing trend over years basically, except for 2014.

As shown in Figure 2, the output of ‘Health services’ increases, while other five sectors appear to have different degrees of output reduction, among which, the losses of ‘Industry’ and ‘Other services’ are relatively more significant. Industrial pollution is the main source of PM_2.5_ pollution [44,45], and hence the reduction in PM_2.5_ concentration implies reduced industrial production capacity.

The decreased input level of individual sectors and the reduced consumption demand of residents, caused by the falling labor force and the lowered overall consumption, go against the sectors’ output increase. Moreover, due to increased medical expenditure, the hot sales of anti-smog products stimulate the development of ‘Health services’. As time goes by, the output losses show a declining trend, and the PM_2.5_-induced economic benefits are gradually shrinking.

## 4. Discussion

In contrast to 2013, the proportion of labor force loss increased in 2014 while medical expenditures decreased. After adopting the ratio of labor force loss and medical expenses into the recursive dynamic CGE model, the losses of all the observed indexes exhibit similar changes in the same direction as labor loss, except for the output of ‘Health services’. This suggests that to some extent, the negative impacts on economic system by labor loss are greater than that of medical costs, which boosts the research results by Yang et al. [46]. 

The average annual loss for 2013–2020 of the regional GDP due to PM_2.5_ pollution is estimated to be 382.83 million RMB in this study. Previously, by using the HCA approach, Fan investigated the economic damage due to SO_2_, NO_2_, and PM_10_ for 2012, and his estimate was 768.58 million RMB (see Section 1.2); by employing the HCA method, Zhao et al. also studied the economic loss due to PM_10_ for 2012, and his estimate was 583.02 million RMB (see Section 1.2). The differences between these estimates reflect the pollution types considered, year of study, and the methods employed, as further elaborated below:

The CGE model can take into account the influence of PM_2.5_ pollution on different economic entities and different industrial sectors. The dynamic CGE model can measure the long-term economic impact of PM_2.5_.

The concentration of the haze pollution tends to decrease over the examined years. While Fan and Zhao estimated the loss for an earlier year (year 2012) due to primarily PM_10_, the paper predicted for future years (2013–2020) due to PM_2.5_.

This study simulates and investigates the changes of different economic subjects responding the health effects of haze under mixed economic conditions by a proposed dynamic CGE model. Dynamic mechanisms have been used to assess the public health value loss of haze pollution from the perspective of economic development. Investigation on the long-term dynamic effects of PM_2.5_ pollution has been introduced in this paper, rather than being limited to the burden of disease through traditional methods such as the HCA and COI. It can reflect the impacts of haze on the various sectors of the economy, and also obtain the impacts of the whole social economy through the model interlocking effect with comprehensive evaluation aspects. However, due to the complexity of the effects of health on the national economy, the study still has some limitations:

(1) This paper does not consider the uneven distribution of population in urban and suburban areas and the fluidity of population, which may lead to a lower assessment result.
(2) Only the impacts on the labor quantity and the medical cost of outpatients and inpatients caused by PM_2.5_ are included in the proposed model, whereas self-medication costs and economic losses caused by declining happiness are not incorporated in the study, implying the estimations could be conservative.
(3) The premature deaths cannot be measured in terms of monetary values and hence are not admitted in the dynamic CGE model, causing lowered estimation for the economic loss.
(4) Limited by the availability of data, the E-R coefficients and baseline concentration in this paper are obtained from other studies.
(5) The ratio of labor force loss and medical expenses involved in the dynamic CGE model are obtained by E-R functions and statistical data rather than via the operation of the CGE model, implying that the complete feedback might not be perfectly achieved in the simulated results.
(6) Due to the complexity of the health system, economic system, and the exclusion of factors such as confounding variables, and the complexity of the ripple effects of health effects on the national economy, there may be existing inevitably uncertainties and limitations in this study and its model.

In terms of social and economic impacts, the proposed CGE model still needs improvement compared to the actual economic system. For example, the CGE model reflects the situation of a completely clearing market, while the actual economic system has inherent inertia and switching costs. Therefore, the calculated results in this paper may be low. Nevertheless, the CGE model for the analysis of the health effects due to haze pollution is still relatively objective, which can effectively reflect the correlation effects of various sectors of the socio-economic system, and follow the objective economic law, i.e., the total input equals the total output; whilst the total supply equals the total demand. Compared with traditional economic loss assessment methods, the evaluation results are more representative for the social and economic losses caused by health effects. This study has proven that PM_2.5_ pollution cannot be ignored considering the economic loss of health impacts, which depicts practical significance and promotion value.

Further analysis can be undertaken in the following aspects:

(1) With complete and accurate data of labor force loss and medical expenses, future research can be conducted leading to more accurate results.
(2) In terms of health damage, the impact of other pollutants in the haze can be considered on human health, such as the long-term effects of SO_2_ and PM_10_ on human health hazards.
(3) The impact of haze on society is complex, while only the impact of population health is investigated in this paper. The impact of haze pollution on other aspects of society such as transportation, agricultural production, tourism, and mental health can also be of concern in the future.

## 5. Conclusions

This paper monetizes the health consequences of haze pollution by using a GA-grey Markov model and E-R functions. Based on the decreased labor force and increase of medical expenses associated with haze pollution, which were investigated and obtained from previous studies in the literature, a recursive dynamic CGE model was proposed. This idea bridges the PM_2.5_ concentration to the dynamic CGE model, yields a more comprehensive assessment for the health economic loss caused by PM_2.5_ pollution, and expands the application areas of the CGE model to the health field.

The empirical analysis results predicted that PM_2.5_ concentration in Beijing decreases during 2013–2020, but still has a serious impact on the public health, including 156,588 (95% CI: 43,335–248,914) premature deaths and 6,397,553 (95% CI: 3,056,828–9,381,570) cases of related diseases. The corresponding accumulated loss of regional GDP due to health effects is estimated to be 3,062.63 (95% CI: 1,168.77–4,671.13) million RMB. In summary, the control of haze pollution, particularly PM_2.5_, is critical for improving health conditions of residents and for reducing damages to the economic system.

## Figures and Tables

**Table 1:** Particulate matter (PM)_2.5_ concentration in Beijing 2013–2017.

Year	2013	2014	2015	2016	2017
PM_2.5_ (μg/m^3^)	89.5	85.9	80.6	73.0	58.0

**Table 2:** The relevant population data in Beijing during 2013–2020.

Year	2013	2014	2015	2016	2017	2018	2019	2020
Mortality rate (‰, deaths per one thousand persons)	4.52	4.92	4.95	5.20	*5.20*	*5.20*	*5.20*	*5.20*
Resident population (million persons)	21.15	21.52	21.71	21.73	*22.00*	*22.27*	*22.55*	*22.82*
Labor force (million persons)	11.41	11.57	11.86	12.20	*12.50*	*12.81*	*13.12*	*13.45*

Note: The estimated values are in italics.

**Table 3:** Related information of health outcomes.

Health Outcome	Coefficient (95% Confidence Intervals (CI))	Incidence	Work Time Loss (Day)
Premature deaths			
All-cause mortality	0.00296 (0.00076–0.00504)	Mortality of each year ^a^	250 ^b^
Hospital admissions			
Respiratory disease	0.00109 (0.00000–0.00221)	0.01619	1.75
Cardiovascular disease	0.00068 (0.00043–0.00093)	0.00855	25.8
Outpatient visits			
Pediatrics (0–14 years old)	0.00056 (0.00020–0.00090)	0.22043	0.5
Inter medicine (15–64 years old)	0.00049 (0.00027–0.00070)	0.66551	0.91
Diseases			
Chronic bronchitis	0.01009 (0.00366–0.01559)	0.00694	1.38
Acute bronchitis	0.00790 (0.00270–0.01300)	0.03800	0.55
Asthma	0.00210 (0.00145–0.00274)	0.01190	0.55

Note: Source: ‘China Health and Family Planning Statistical Yearbook (2014)’, Huang and Zhang [34], Wang et al. [35]. ^a^ The mortality data are presented in Table 2. ^b^ Excepting the eleven-day public holidays and fifty-two weekends (Saturday and Sunday), there are 250 working days in a year.

**Table 4:** Social accounting matrix (SAM) in Beijing in 2012 (hundred million RMB).

		Commodity	Activity	Factor		Household	Government	Saving-Investment	Total
				Labor	Capital				
Commodity			34,632			6203	4452	7410	52,697
Activity		52,697							52,697
Factor	Labor		9117						9117
	Capital		5987						5987
Household				9117	5987				15,103
Government			2961			1490			4452
Saving-Investment						7410			7410
Total		52,697	52,697	9117	5987	15,103	4452	7410	147,462

**Table 5:** The predicted results of Grey Markov (GM (1,1)) and the state division of the residual series.

Year	2013	2014	2015	2016	2017
Actual value (μg/m^3^)	89.5	85.9	80.6	73.0	58.0
Predicted value (μg/m^3^)	89.5	88.1	78.2	69.4	61.6
Residual (μg/m^3^)	0.0	−2.2	2.4	3.6	−3.6
State	*d*_2_	*d*_1_	*d*_3_	*d*_3_	*d*_1_

**Table 6:** The predicted results of the traditional grey Markov model and genetic algorithm (GA)-grey Markov model.

Year	Actual Concentration (μg/m^3^)	Grey Markov Model (μg/m^3^)		GA-Grey Markov Model (μg/m^3^)	
		Predicted Concentration	Residual	Predicted Concentration	Residual
2013	89.5	89.5	0.0	89.5	0.0
2014	85.9	85.1	0.8	86.1	−0.2
2015	80.6	81.2	−0.6	80.2	0.4
2016	73.0	69.4	3.6	69.4	3.6
2017	58.0	63.1	−5.1	62.6	−4.6

**Table 7:** The evaluation results of the models. Mean squared error (MSE).

Model	Average Relative Error	Relational Grade	Posteriori Error Ratio	Small Error Probability	MSE
GM (1,1) model	0.0334	0.5095	0.1177	1.0000	7.303
Grey Markov model	0.0308	0.6637	0.1770	1.0000	7.994
GA-grey Markov model	0.0272	0.7007	0.1741	1.0000	6.873

**Table 8:** The predicted results of PM_2.5_ concentration for Beijing.

Year	2018	2019	2020
Predicted value (μg/m^3^)	55.2	49.3	43.7

**Table 9:** Two conducting variables of the computable general equilibrium (CGE) model.

Year	Ratio of Labor Force Loss (‰)	Medical Expenses (Million RMB)
2013	0.82 (0.32–1.23)	1113.21 (290.94–1881.62)
2014	0.83 (0.32–1.25)	1088.66 (284.27–1842.69)
2015	0.77 (0.30–1.17)	1032.74 (269.32–1751.69)
2016	0.71 (0.27–1.08)	939.38 (244.49–1598.08)
2017	0.56 (0.21–0.87)	760.34 (197.15–1301.17)
2018	0.53 (0.20–0.82)	733.41 (190.02–1256.50)
2019	0.47 (0.18–0.73)	664.72 (171.97–1141.51)
2020	0.42 (0.15–0.65)	597.91 (154.45–1029.06)

**Table 10:** Macro-economic effects of PM_2.5_ pollution (million RMB).

Year	Residents’ Income	Government Revenue	Residents’ Consumption	Government Consumption	Regional Gross Domestic Product (GDP)
2013	−748.87	−148.70	−307.58	−148.70	−456.27
2014	−776.84	−154.25	−319.06	−154.25	−473.31
2015	−741.34	−147.20	−304.48	−147.20	−451.69
2016	−694.00	−137.80	−285.04	−137.80	−422.85
2017	−567.41	−112.67	−233.05	−112.67	−345.72
2018	−548.40	−108.89	−225.24	−108.89	−334.13
2019	−499.10	−99.10	−204.99	−99.10	−304.09
2020	−450.64	−89.48	−185.09	−89.48	−274.57

Note: ‘−’ means decrease.
